# Citizens under the umbrella: citizenship projects and the development of genetic umbrella organizations in the USA and the UK

**DOI:** 10.1080/14636778.2019.1693889

**Published:** 2020-03-06

**Authors:** Koichi Mikami

**Affiliations:** Faculty of Science and Technology, Keio University, Kanagawa, Japan

**Keywords:** New Genetics, Genetic Citizenship, Umbrella Organization

## Abstract

Social scientists have observed previously that patient support groups began to have significant influence over both research and clinical services of medical genetics in the early 2000s. This observation led to the idea of genetic citizenship, suggesting that the active participation and intervention of patient support groups in the rapidly growing field of medicine marked the emergence of a new form of citizenship. To understand how this citizenship emerged, this paper examines the development of umbrella organizations of genetic support groups in the USA and the UK. The historical analysis demonstrates that the ways in which these organizations developed differ considerably, and that their visions and activities reflected the different structural and cultural organizations of medical genetics in their respective countries. By recognizing the early work of these organizations as citizenship projects, this article argues that they helped rather different forms of genetic citizenship to emerge in the two countries.

## Introduction

In this article, I study the rise of a form of citizenship associated with advances in medical genetics – that is, genetic citizenship. I do so through a historical analysis of the development of two organizations: the Alliance of Genetic Support Groups (AGSG, now the Genetic Alliance) in the USA and Genetics Interest Group (GIG, now the Genetic Alliance UK) in the UK. These umbrella organizations – so called because they provide common organizational structures linking a number of different genetic disease organizations – were both established in the 1980s, and have since been active in the field of medical genetics in and beyond their respective countries. Earlier social studies of medical genetics have noted the importance of these organizations (e.g. Heath, Rapp, and Taussig 2004; Kerr 2003; Parthasarathy 2004, 2007; Petersen 2002; Raz 2010; see also Harper 2008), but little has been done to explore how they differed from one another. This paper shows that attention to their differences helps to highlight the different forms of genetic citizenship that emerged in the two countries at the beginning of the twenty-first century.

The concept of genetic citizenship first appeared in social studies of medical genetics in the early 2000s (e.g. Heath, Rapp, and Taussig 2004; Kerr 2003; Petersen 2002; Rose and Novas 2005; Taussig 2007). Social scientists were at that time voicing concerns that advances in genetics were re-introducing eugenic practices under the guise of informed patient choice (Cunningham-Burley and Kerr 1999; Kerr 2003; Kerr and Cunningham-Burley 2000; Kerr, Cunningham-Burley, and Amos 1997; 1998; Petersen 1998; 2002). Also in that context, however, it was observed that rights and responsibilities were being re-distributed between medical and political authorities on the one hand and patients and their families on the other (Novas and Rose 2000; Rapp and Ginsburg 2001; Taussig 2005). The concept of genetic citizenship reflected the recognition that patient support groups played key roles in facilitating this re-distribution (Heath, Rapp, and Taussig 2004; Taussig 2007).

Personal biological attributes have for long been part of the conception of what it means to be a citizen. That conception was originally framed primarily in relation to the idea of the nation state: “[c]itizenship was fundamentally national” (Rose and Novas 2005, 439; see also Anderson 1983 [2006]; Ong 1999). While national connotations persist (cf. Sigurdsson 2001), new conceptions of citizenship are in contrast seen to be associated with other kinds of communities – including those imagined through knowledge of one’s genetic make-up, and through a shared interest in challenging the fate that that make-up implies (Novas 2006, 2007, 2008; Novas and Rose 2000; Panofsky 2011; Rabinow 1996; Raz 2010; Rose 2007).

The present study is informed by Rose and Novas’ idea of “citizenship projects,” by which they mean “the ways in which authorities thought about (some) individuals as potential citizens, and the ways in which they tried to act upon them” (2005, 439). An important question to ask in applying this idea to analysis of the rise of genetic citizenship is: who were the authorities who shaped the conception of what it means to be a genetic citizen? It has been argued that patient groups are increasingly assuming such an authoritative position, alongside their research and policymaking partners (e.g. Novas 2006, 2007, 2008; Panofsky 2011; Rabeharisoa 2003; Rabeharisoa and Callon 2004; Stockdale and Terry 2002). Building on this argument, I examine how the roles of genetic citizens were understood in the early days of the AGSG and GIG, and discuss how the work of these organizations enabled these particular versions of citizenship to become accepted in their respective countries.

In the next section, I first present a brief review of the literature on the new genetics, and contrast it with that on the active roles of patient organizations working in that field. Following a short section on methodology, I then present detailed histories of the AGSG and GIG. I show that the AGSG was developed as a venue through which patient organizations could share their knowledge and skills as resources with which they engage with professional genetic services. GIG, in contrast, was formed primarily to represent a unified voice of patient organizations in policy debates. In the discussion, I focus on the question of what their contribution to the citizenship project was, and argue that different genetic citizenship projects emerged in the two countries because the national specificities of healthcare and genetic services – what Parthasarathy (2007) calls the national “toolkits” - led families with genetic diseases to take rather different approaches in order to improve their circumstances.

## Patient support groups in the new genetics era

At the turn of the century, the term “the new genetics” was criticized by social scientists as a rhetorical device serving to obscure a resurgence of eugenic thinking (e.g. Cunningham-Burley and Kerr 1999; Kerr 2003; Kerr and Cunningham-Burley 2000; Kerr, Cunningham-Burley, and Amos 1997, 1998; Petersen 1998, 2002). In medical discourse, the term was often associated with ideas about patients’ “right to know” and “informed choice,” which both presumed the neutrality of medical genetics experts (Arribas-Ayllon, Sarangi, and Clarke 2011). As a number of social scientists observed, however, medical geneticists tended to suppose that rational properly informed individuals would choose where possible to eliminate genetic “errors” from their families, and thereby from society. In effect, social scientists contended, the term served to devolve responsibility for eugenic decisions from geneticists to affected individuals and families (see Hallowell 1999; Kerr 2003; Kerr and Cunningham-Burley 2000; Lemke 2007; Petersen 1998; Rapp and Ginsburg 2001).

It was David E. Comings, then editor of the *American Journal of Human Genetics*, who first used the term “the new genetics” in 1980 (Weatherall 1991; see also Arribas-Ayllon, Sarangi, and Clarke 2011). Comings’ invocation of a “new” genetics was meant to convey his excitement with both a shift in analysis of human genetic disease from biochemical to molecular and the new medical possibilities this opens up (see Comings 1980). Molecular techniques indeed facilitated the cloning of the genes for several rare genetic conditions providing valuable clues to understanding their biochemical basis in the 1980s (see Harper 2008). As Weatherall wrote in 1991, molecular genetics offered medical geneticists new tools that, in the long run, would “broaden the scope of the genetic analysis of human disease to encompass the cell and molecular biology of a variety of the major killers of western societies” (1991, 2; see also Childs 1986; Watson 1990).

The very prospect that excited Comings, however, caused considerable concern to some historians and social scientists in the early 1990s (e.g. Duster 1990; Kelves 1992; Lippman 1991, 1992; Nelkin and Tancredi 1994; Paul 1994). Lippman (1991, 1992), for instance, introduced the term “geneticization” to denote the trend to privilege molecular genetics as the way to understand health and disease. Yet advances in medical genetics only strengthened this trend. The first significant milestone was the identification of the gene for Huntington disease in 1993. The presence of its pathogenic variant means that an individual will with certainty develop this devastating late-onset disease in the future, rendering the person genetically diseased however healthy he or she may be at present (Novas and Rose 2000). The trend further continued with identifications of genetic risk factors for several more common diseases, including breast and ovarian cancer, schizophrenia and diabetes (Hallowell 1999; Hedgecoe 2001, 2002). These discoveries also fueled growing enthusiasm for gene therapy to correct such genetic “errors” in this new genetics era (see Martin 1999; Stockdale 1999).

It was, however, also in this context that social scientists observed the emergence of “innovative forms of public intervention and participation” (Glasner, Rothman, and Cambrosio 1999, 7). Although significant gaps existed between patients and medical researchers in the late 1990s (e.g. Stockdale 1999), from the early 2000s researchers reported a number of cases where a patient support group successfully influenced health policy and/or medical research (e.g. Gibbon 2008; Heath, Rapp, and Taussig 2004; Rabeharisoa 2003; Rabeharisoa and Callon 2004; Stockdale and Terry 2002; Taussig 2005, 2007). While collective action by patients and their families was becoming increasingly common across various areas of medicine (see Allsop, Jones, and Baggott 2004), action around genetic conditions in particular came to be seen as exemplifying what Rabinow (1996) called “biosociality.”

Particularly remarkable among such cases were the work of the muscular dystrophy association in France (Rabeharisoa 2003; Rabeharisoa and Callon 2004) and that of Sharon and Patrick Terry in the USA (Novas 2006, 2007; Rose and Novas 2005; Stockdale and Terry 2002; Taussig 2005, 2007). The French muscular dystrophy association – the AFM – was created in the late 1950s with the aim of supporting research and ultimately finding a cure for the disease (Bach 1998). Its success in television-based fundraising in the early 1990s gave it the resources to partner on a more equal footing with scientists (Rabeharisoa 2003; Rabeharisoa and Callon 2004). Among other things, the AFM emphasized the value of the knowledge that only patients and their families possess as a result of living with the disease. In contrast, the Terrys leveraged patients’ own bodies to influence research into a rare genetic skin disorder called pseudoxanthoma elasticum (PXE). By founding a patient organization called PXE International in the mid-1990s and developing a blood and tissue bank and a patient registry (Terry and Boyd 2001; Stockdale and Terry 2002), they were able to secure control over scientists’ access to biological materials, and thereby to set the terms of collaboration (Terry 2003; Novas 2006, 2007). The research resulted in the identification of the PXE gene in 2000, and the Terrys’ work became recognized as a model for other patient support groups, which some called “the PXE model” (Heath, Rapp, and Taussig 2004; Taussig 2005, 2007).

Panofsky (2011) observes that nowadays patient groups concerned with genetic diseases tend to make use of whatever resources are available to them – including but not limited to financial and biological resources – to foster their ability to work with scientists and build a relationship with those interested in studying their diseases. At the same time, patient groups are increasingly recognized by health policy experts as sources of valuable information representing the views of patients and their families (Jongsma *et al*. 2018; Baggott and Jones 2018). They therefore function as an organizational platform for patients and their families to involve themselves in research and policymaking. And through such involvement, patients and families also re-shape their identities and redefine what it means to possess the genetic make-up that brought them together in the first place (see Heath, Rapp, and Taussig 2004; Novas 2008). It is this active, collective, biologically informed engagement with science and policy that Rose and Novas call “biological citizenship,” of which genetic citizenship is a specific subcategory. As they stress, moreover, “an analysis of biological citizenship cannot merely focus upon strategies for ‘making up citizens’ that are imposed from above” (2005, 441). Therefore, any analysis of genetic citizenship must take account of how patients and their families defined their own roles in shaping the new genetics era.

At the same time, it is important to note that patients and families had to work with the particular resources available to them, and did so within particular organizational and political contexts. As I demonstrate in this article, both the AGSG and GIG played a vital role in enabling patients and their families to engage actively in improving the genetic services available to them, not least by helping to create imagined communities of genetic citizens with whom they could work. Yet the organization and culture of the medical genetics services and institutions with which they engaged in their respective countries differed in important ways (cf. Parthasarathy 2005, 2007). Consequently, the forms of action available to them, and ultimately the aims they sought to achieve, also differed significantly. In effect, as this article shows, genetic citizenship took rather different forms in the USA and the UK.

## Methodology

This study is based on two kinds of historical sources relating to the two organizations: first, documentary sources, both published and unpublished; and secondly, a series of oral history interviews. I began with a preliminary review of relevant documents identified through a search of published and archival literature. Of these, the proceedings of two conferences held in the mid-1980s (Weiss, Bernhard, and Paul 1984, 1986) proved particularly informative of the contexts and motives behind the establishment of the AGSG. Although there was no such record for the British organization, I was able to draw on a document entitled *A brief history of GIG* (GIG 2006), which used to be available on its website, and also its internal documents, including minutes of early board meetings. From a preliminary review of these documents, it became apparent that key questions to understand the emergence of genetic citizenship in the early twenty-first century included how the relationship between medical geneticists and patients and families affected by genetic diseases developed and how the establishment of the two organizations influenced that development.

While the documentary sources went some way to answer these questions, oral history interviews with key participants provided additional information. Most interviewees were identified from their presence in the documentary sources, but some were contacted because other interviewees suggested them to be key informants – i.e. through a snowball-sampling approach. The 10 interviews with those who involved in the establishment of the AGSG or who were active in research and/or practice of medical genetics in the USA at that time were conducted between January and March 2015. The equal number of interviews with their British equivalents, i.e. individuals active in GIG in its early days and medical geneticists who had close contact with them, were conducted between August and December 2015. An additional interview with a person knowledgeable about the activities of patient support groups at a European level in the 1990s was conducted in the Netherlands in February 2016. As is standard methodology in oral history, the aim of these interviews was not to generate “objective” data about past events, but to elicit respondents’ personal memories, reflections and narratives of those events, which are often left out in documentary sources (Portelli 1981).

Analysis of the documents and interviews proceeded in the first instance by constructing parallel narrative accounts of the development of the two organizations, focusing particularly on what the sources together enabled me to say about the changing relationship between medical geneticists and patients and families affected by genetic diseases in the two countries. Attention was also paid to how the organizations responded to developments in the science of medical genetics. Triangulating between the documentary and oral history sources served to ensure that these narratives were as far as possible accurate in their depiction of historical events and interactions, while the content of those sources was analyzed to elucidate how the historical actors experienced those interactions, and the meanings and intentions they ascribed to them. The following two sections present these accounts in some detail, highlighting moments and episodes that are particularly informative about the distinctive ways in which patient organizations in the two countries sought to make practices of medical genetics work for them.

## The Alliance of Genetic Support Groups

The AGSG was founded by clinical social worker Joan O. Weiss in 1986. In the late 1960s, Weiss had joined the Medical Genetics Clinic at the Johns Hopkins Hospital, led by prominent medical geneticist Victor A. McKusick. Although the potential importance of social work in medical genetics had been suggested (e.g. Schild 1966), few clinical social workers were employed at genetics clinics at that time, and Weiss was among the first. She was appointed, not because McKusick recognized the value of having a social worker in his team, but because it was a condition for receiving a research grant. Weiss, for her part, had little understanding of genetics at that time, and initially confused it with geriatrics (interview US06).

Weiss’s main responsibility at the clinic was to provide support in dealing with psychosocial problems to the families with genetic conditions who came to see McKusick from all over the country. McKusick had a particular research interest in the heritable disorders of the bones and connective tissues (see Harper 2008; Lindee 2005), including dwarfism, osteogenesis imperfecta, and Marfan syndrome. As McKusick had a close relationship with the Little People of America, a support group for persons of short stature and their families, since 1965 (Francomano and Rimoin 2012; McKusick 2006), Weiss quickly became involved with this group too. However, she thought more could be done to support the members than simply organize annual conventions, which she characterized as opportunities “for friendship, dating and eventual marriages” (1977, 59). Consequently, from the early 1970s, she and McKusick organized an annual Short Stature Symposium at Johns Hopkins, including educational workshops to build confidence and social coping strategies among those affected (Weiss 1977). This experience of working closely with a patient support group would inform her later inception of the AGSG.

### Changing working environment at Johns Hopkins

Meanwhile, medical genetics more generally underwent important changes through the 1970s. First, developments of genetic screening led to changes in the relationship between medical geneticists and patient support groups. In 1972, following the roll-out of newborn mass screening for phenylketonuria (see Lindee 2005; Paul and Brosco 2013), the State of Maryland passed an act to introduce screening of sickle cell anemia. The act was quickly repealed following the recommendations of a committee consisting of medical professionals and consumer representatives, and instead a statutory Commission on Hereditary Disorders, again comprising professionals and consumers, was convened to oversee management of genetic diseases in the State (Holtzman, Lapides, and Clarke 1974; Holtzman 1980). The Commission provided an opportunity for professionals to educate consumers – mostly representatives of patient support groups – about genetics (interview with US02). Around the same time, Michael Kaback at Johns Hopkins developed a carrier-screening program for Tay-Sachs disease in collaboration with leaders of local Jewish communities (Kaback 2014; Wailoo and Pemberton 2006). Medical geneticists thus started to recognize the merit of working with laypersons, while also seeing a need to educate them properly about the science of genetics.

Second, genetics clinics across the country were becoming populated with a new kind of professionals – non-medically trained genetic counselors. When the term “genetic counseling” was coined in the 1950s, it was considered as a job for professionals with a medical degree or at least a PhD (e.g. Reed 1955; Epstein 1973). However, the first master’s degree program for genetic counselors was launched in 1969, and several more followed (see Stern 2012). In 1975, an *ad hoc* committee of the American Society of Human Genetics (ASHG) recommended that genetic counseling should not just concentrate on explaining the medical aspect of genetic disorders but should also attend to the psychosocial impacts of genetic diagnosis (ASHG Ad Hoc Committee on Genetic Counseling 1975). While the involvement of medical professionals was still considered essential, genetic services were increasingly seen as a team effort, in which clinical social workers like Weiss also had an important role to play.

Locally this shift in the constitution of medical genetics was manifested with the appointment of two new staff members to the Johns Hopkins Hospital’s Medical Genetics Clinic. In 1977, medical geneticist Reed E. Pyeritz joined McKusick’s team, and started seeing families with Marfan syndrome (Pyeritz 2012). In the same year, he attended the annual convention of the Little People of America, which he describes as an “eye-opening” experience (Pyeritz 2012, 73). He went on to found a support group for families affected by Marfan syndrome, with three principal aims: providing mutual psychosocial support among its members, supporting medical research on the syndrome, and educating physicians and newly diagnosed patients (interview US04). The second appointment was genetic counselor Barbara A. Bernhardt, who joined the clinic as its first non-medically trained genetic counselor in 1980. Previously the clinic had “genetic assistants,” each of whom looked after patients and families with a specific condition on their hospital visit. As Bernhard replaced them and started interacting with families with different genetic disorders, she began to recognize that they faced many issues in common. With Weiss’s encouragement, she also built good relationship with patient support groups (interview US09). Working with these colleagues reinforced Weiss’s view that not only social workers but also patient support groups could play an important role in improving the team effort of genetic services (interview US06).

### Building the coalition of genetic support groups

Late in 1982, Weiss approached the March of Dimes Birth Defects Foundation – a charity that had supported McKusick’s work on various occasions – with a proposal for a conference to explore the partnership between medical professionals and patients and their families. The Foundation’s then Vice President of Professional Education, Beverly Raff, whose background was in nursing, was keen on Weiss’s idea, and agreed to support the event (see Raff 1990). Weiss also managed to secure funding from the US Department of Health and Human Services, and the conference *Genetic Disorders and Birth Defects in Families and Societies: Toward Interdisciplinary Understanding* was held in Baltimore in April 1983 (Weiss, Bernhard, and Paul 1984). Pyeritz and Barnhardt were members of the planning committee, as was Barton Childs, another leading medical geneticist at Johns Hopkins with a long-standing interest in educating physicians and laypersons about genetics (interview US02 and US10; see also Childs 1999).

While Weiss’s principle agenda for the conference was to demonstrate the value of the partnership between medical genetics experts and the patients and their families they serve, she also sought to bring other voices into the discussion (interview US06). Reflecting the ongoing changes in medical genetics, she framed the conference theme as fostering an “interdisciplinary” approach to genetic services – inviting, for example, geneticists Edward Hsia and Arno Motulsky, clergyman Robert Baumiller and Eugene Lipman, legal scholars Alexander Capron and Phillip Reilly, genetic counselors Joan Burns and Joan Marks, and social work scholars Sylvia Schild and Rita Beck Black, as well as parents of patients with genetic disorders. By getting them all in the same room, the event successfully signaled that genetic services would be better delivered through interdisciplinary teamwork (McKusick 1984; Childs 1984). Weiss’s move to highlight the interactional aspects of genetics services was also welcomed by medical geneticists, whose time-consuming counseling work was insufficiently reimbursed, weakening their financial position within their hospitals (Interview US04; Pyeritz 1984; see also Pyeritz, Tumpson, and Bernhardt 1987).

Soon after the conference, Weiss started thinking about building a coalition of patient support groups, partly inspired by discussions in the session “Family Support Groups: Setting Goals,” in which support groups learnt from each other’s work. Particularly significant was the talk by Mary Ann Wilson (1984), a member of the National Neurofibromatosis Foundation, mentioning her involvement in the establishment of the National Organization for Rare Disorders, a coalition of around 20 rare disease organizations founded by Abbey Meyers in 1983. Meyers’s success in building this coalition encouraged Weiss to pursue a similar strategy for genetic disorders (interview US06), and in June 1985 she organized a conference on *Genetics Support Groups: Volunteers and Professionals as Partners* (Weiss *et al*. 1986). Key to the conference was a session entitled “Is There a Network of Genetic Support Groups in Our Future?” chaired by Weiss’s trusted colleague Pyeritz and a parent of a patient with Down syndrome (interview US06). The session concluded that while individual support groups might best serve some purposes, a coalition could be beneficial for others. Consequently, a steering committee consisting of Weiss, Pyeritz, and representatives from a number of patient support groups was organized to work towards the establishment of a coalition (Pyeritz and Weigle 1986). In early 1986, the AGSG was founded, with Weiss as its executive director. For the first two years she continued to work at Johns Hopkins but eventually decided to commit to the AGSG full-time.

### The coalition’s relationship with professionals

The AGSG’s early work reflected the goals set out at the 1985 conference, including educating professionals and the public about genetic disorders, sharing technical and administrative skills for running support groups, and promoting partnership between individual groups and professionals (see Pyeritz and Weigle 1986; Weiss 1989; Macketa and Weiss 1994). Policy advocacy on issues such as federal support for genetic services and medical research was also suggested, but that was not Weiss’s strong point (interview US06). Instead, drawing on her experience in clinical social work, she introduced a toll-free helpline for newly diagnosed patients, staffed by a social worker. The AGSG maintained a directory of patient support groups for genetic disorders, so was able to put such patients in touch with an appropriate group, and where no group existed, the AGSG offered advice on how to set one up (see Weiss and Mackta 1996). The service was also frequently used by genetic counselors to find the right group for their patients (interview US07). These groups functioned as important channels of information for individual families to evaluate the serves they received at hospitals, as well as providing alternative support services where the professional services fell short. Individual members of the groups were in return expected to utilize their experiences as shared resources of the community that they were part of.

In 1996, Weiss stepped down as the AGSG’s executive director for family reasons. She did so at the start of a difficult time for the organization. The late 1990s saw a growing number of initiatives to educate professionals and the public about genetics, many spun off from the Ethical, Legal, and Social Implications research program of the Human Genome Project. The Internet also made information about genetic disorders and patient support groups much more accessible than previously. Consequently, there was less demand for the services that the organization provided (interview US08). Before her departure, Weiss had begun to take a more active role in policy advocacy, notably around genetic discrimination (Lapham, Kozma, and Weiss 1996), and her successor as executive director, Mary E. Davidson – also a social worker by training – continued that work, as well as changing the name of the organization to the Genetic Alliance to reflect this more political identity. But it was the appointment of Sharon Terry as president in 2002 and later CEO that really marked a change of direction (interview US06 and US07).

Terry’s decision, as founder-director of PXE International, to focus on promoting research had initially prompted criticism from other patient organizations, who saw it as a departure from their proper role of providing peer support (interview US08). With the identification of the PXE gene in 2000, however, the group came to be seen as a successful model for patients and families to “participat[e] in research as active and critical collaborators as well as donors of genetic material” (Terry and Boyd 2001, 178; see also Novas 2006, 2007; Heath, Rapp, and Taussig 2004; Terry and Boyd 2005, 2007). From 2002, Terry made this research-partnership model central to the work of the Genetic Alliance, establishing a patient registry and biobank in 2003 (Terry 2003), while older services such as the telephone helpline were replaced by an online wiki page. In effect, Terry expanded the organization’s existing citizenship project: where previously that project had revolved around mobilizing patients’ and families’ knowledge and experience to stand alongside clinicians as partners in providing genetic services, under Terry it came to involve using patients’ genetic material to claim a role as equal partners in genetic research (see Black and Weiss 1988; Lin *et al*. 2003).

## The Genetics Interest Group

While the establishment of the AGSG was due largely to the vision of a single individual, the creation of GIG owed more to a collective decision by a number of patient support groups. Individual initiative helped to lay the groundwork, however – in this case that of a medical geneticist, Rodney Harris, at the University of Manchester.

Harris was recruited to the genetics clinic at the Manchester Royal Infirmary in 1968. He soon faced rapid growth in demand for his services with the development of prenatal testing for Down syndrome and subsequently neural tube defects, boosted by the legalization of abortion under the UK Abortion Act of 1967 (Leeming 2005). Harris was not the only geneticist struggling to meet the demand for testing. As a result of concerted professional pressure, from 1974 regional genetic services were established as part of the National Health Service (NHS) (Coventry and Pickstone 1999; Fitzsimmons *et al*. 1982; see also Leeming 2005). The initiative was led by Cedric O. Carter, a medical geneticist at the Institute of Child Health (ICH) in London, who had become the first consultant advisor in medical genetics to the Chief Medical Officer at the UK Department of Health and Social Security (DHSS).

### Changing technical environment at regional genetic services

In 1982 Harris succeeded Carter in that post (see Harris 1997). He found that, while clinical genetics had been listed as a clinical specialty by the DHSS (Reynolds and Tansey 2010), the Department was doing little to support medical genetics at that time (interview UK08). Resourcing fell far short of what geneticists felt was necessary to provide adequate services, and Harris worried that this would lead to crisis, particularly as it became apparent that services would need to expand further to take advantage of new molecular techniques (Johnston and Harris 1983).

In this context, in 1984 Harris persuaded the DHSS to start a Special Medical Development program in clinical genetics to evaluate the importance of molecular genetics for NHS clinical services (interview UK08). The program was conducted at three regional services – Cardiff, Manchester, and the ICH – and its interim report appeared in 1987, recommending molecular genetics laboratories be integrated into genetic services (DHSS 1987; see also Harris 1987; Harris and Wertz 1989; Harris *et al*. 1989). The report’s recommendations were endorsed by an expert forum hosted by the health policy thinktank the King’s Fund in December 1987, which argued that “the good quality services at present deployed in [the three] regions should be available throughout the country” (Anonymous 1987, 1553). To facilitate this, Harris (1988) called for better coordination among genetic services across the country.

Advances in molecular genetics, however, posed challenges as well as opportunities to medical geneticists. One of these involved the ethics of pre-symptomatic testing of Huntington disease. In 1983 researchers identified a tightly linked DNA marker called G8, making it possible in principle to conduct tests to predict which individuals within an affected family were likely to develop the condition. In the UK, Peter S. Harper, a medical geneticist at Cardiff, began assessing its clinical utility (Harper and Sarfarazi 1985; Harper *et al*. 1985). Harris urged caution, however, writing an article with psychiatrist David Craufurd that highlighted the possible risks associated with a predictive test for an untreatable degenerative disease, and stressed the need to study its “psychological and social consequences […] to evaluate [its] benefits and hazards” (Craufurd and Harris 1986, 251). Given these concerns, Harper’s group shifted their focus to consider the possibility of using the test, not to identify those who would go on to develop the condition, but to enable expectant parents to determine whether or not their fetus might be at risk – a so-called exclusion test (Quarrell *et al*. 1987).

Children of parents with Huntington disease considered this caution unnecessary, however, arguing instead that they should have the option of finding out if they themselves had inherited the fatal gene variant (interview UK10). Many were already experiencing anxiety, simply from knowing that they had a 50% risk of developing the disease that had so dramatically changed their parents’ live (see Korer and Fitzsimmons 1985; Wexler 1979). In 1987, Shirley Dalby of the Association to Combat Huntington’s Chorea contacted Harris to demand a re-appraisal of what she considered a “paternalistic” approach. Harris responded that patients should present a unified voice if they wanted a change of policy (interview UK10).

The exchange seems to have alerted Harris to the possibility that, suitably coordinated, patients’ voices could help to drive policy, not just on Huntington disease, but on the provision of genetic services more generally. Later in the same year, he wrote:

the DHSS and the NHS regions require unambiguous evidence of need from consumers before major developments can be approved. Ironically, patients with genetic diseases may be the least able to express their needs, and the pleas of individuals at risk of relatively rare genetic diseases will go largely unheard. If consumers want genetic services a coordinated approach from an association of all the genetic organisations is essential, and this should include factual data on the benefits of the new genetics for their members. (Harris 1987, 350)

Just as he saw a need for better coordination among genetic services across the country, so he also saw the value that a coordinated patient advocacy could bring to efforts to improve genetic services.

### Bringing patient support groups under the umbrella

In May 1987, Dalby organized an informal meeting with individuals from several patient support groups that she knew of, including Ann Hunt of the Tuberous Sclerosis Association, Dee Heaps of the Tay Sachs & Allied Diseases Association, Lesley Greene of the Research Trust for Metabolic Diseases in Children, and Christine Lavery of the Society for Mucopolysaccharide and Related Diseases (the MPS Society) (GIG 1989b, 2006). Some of the participants had also been involved in the fight against Enoch Powell’s Unborn Child bill of 1985, which aimed to ban embryo research. Armed with this experience, the participants supported the idea of building an association to represent the unified voice of patients and their families concerned about genetic diseases, and several subsequent meetings were held to refine the idea (interviews UK03, UK05 and UK10).

In May 1988, Hunt and Lavery made a poster presentation at the Clinical Genetics Society (CGS) meeting, setting out their intention to establish the association (interview UK03; see also GIG 1989b). Most medical geneticists welcomed the idea. They knew the importance of patients’ voices from their earlier fight against the Unborn Child bill and more recently against David Alton’s bill of 1988, which attempted to set an 18-week legal limit on abortion, rendering termination after genetic testing impossible (interview UK04; see also Harris and Wertz 1989). Later in that year, a steering group was formed, and GIG was founded in early 1989.

**Figure 1.**
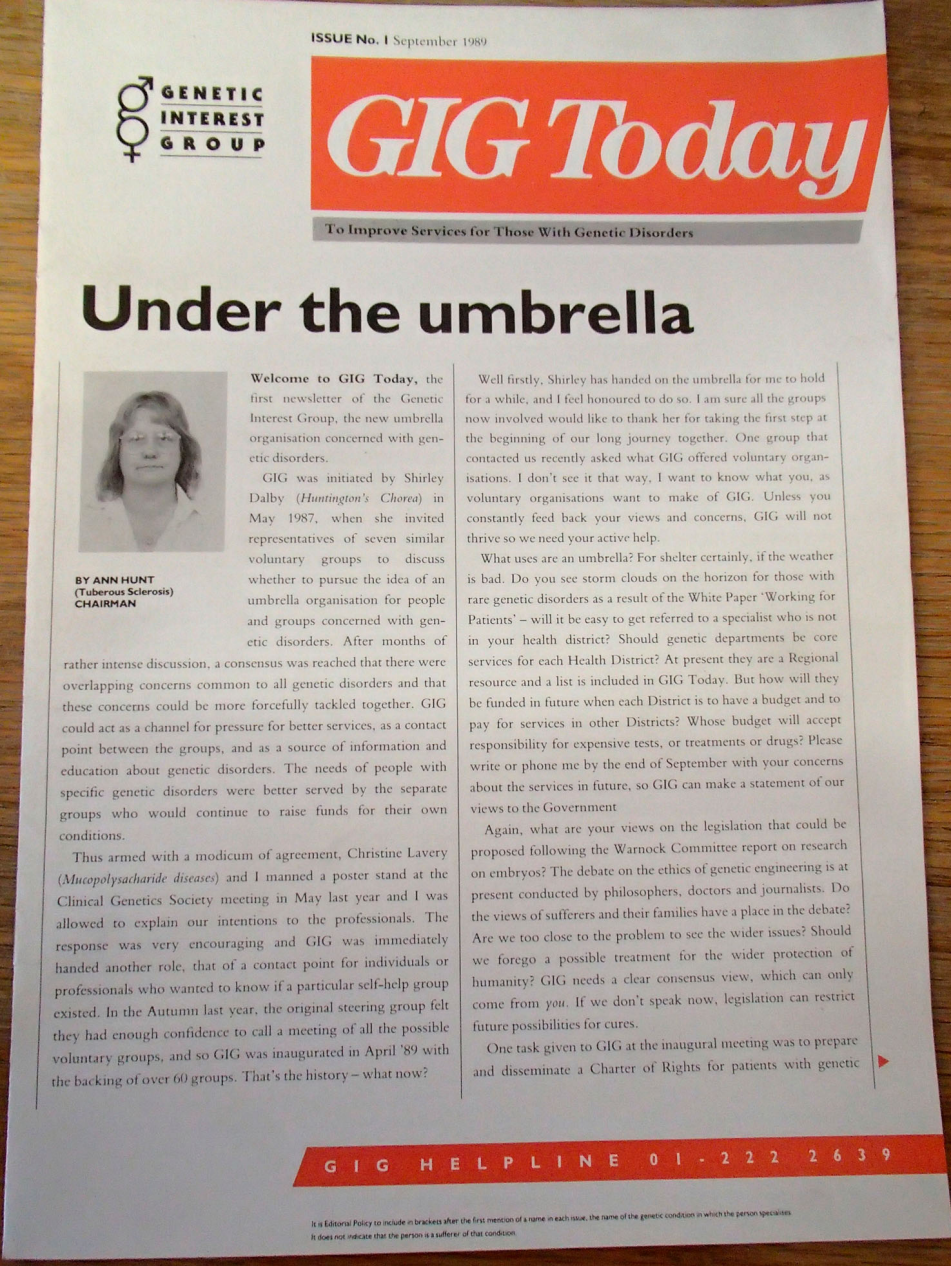
“Under the Umbrella” was the heading Ann Hunt chose in her welcome note of the first *GIG Today* (GIG 1989b).

GIG’s inaugural meeting was held at St Mary’s Hospital, London in April 1989. In the morning, Harris, Michael Patton, a medical geneticist at St. George’s Hospital in London, and Ian A.F. Lister Cheese, an officer of the DHSS, each gave a talk welcoming the founding of the association (GIG 1989e). In the afternoon, Hunt and medical geneticist Susan Chamberlain of the Friedreich’s Ataxia Group were elected the organization’s chair and vice chair respectively. The constitution stated that GIG’s aims were to represent the interest of patients and their families, to promote medical and public awareness of genetic diseases, and to monitor statutory provision and promote the development of genetic services in the country (GIG 1989a). Genetics Helpline – a telephone line through which individuals suffering from a genetic disorder could seek help from their fellow patients – was made available by Lavery through her contract with Contact A Family (interview UK03). By the time the first issue of the newsletter *GIG Today* was published in September 1989 (Figure 1), 60 organizations had signed up to as fee-paying members (GIG 1989b).

### The coalition’s relationship with professionals

GIG’s early work relied on the limited resources made available by its member organizations – including their time, experiences and professional networks. At the first board meeting in May 1989, it was agreed that medical geneticists would be invited to serve the organization’s medical advisory board (GIG 1989c). In addition to Harris and Patton, who spoke at the inaugural meeting, Martin Bobrow, Michael Connor, Nick Dennis, Malcolm Ferguson-Smith, Norman Nevin, David Weatherall and Bob Williamson all accepted its invitation (GIG 1989d). Close association with these prominent medical geneticists was considered to “give credibility” to the new organization; if needed, they were also available for professional advice (interviews UK03 and UK09). Another medical geneticist who supported the work of GIG was Marcus E. Pembrey at the ICH (interviews UK01, UK03 and UK10). Because he took over from Harris the post of consultant advisor to the Chief Medical Officer in 1989, he found it inappropriate to be “aligned” with the organization and did not become its advisor initially (interview UK04). Yet he not only worked with GIG and its members individually but also helped the organization to enhance its credibility by naming it as a useful source of information on some official occasions (e.g. DoH 1993).

GIG made several important developments in the early 1990s, including the appointment of Alastair Kent as its director. Although members of the management committee, particularly chairperson Ann Hunt, were passionate about GIG’s work, its activities, which relied on their voluntary work, remained responsive and somewhat haphazard, and the member organizations were not always well informed about them, inevitably leaving some dissatisfied (interviews UK03 and UK05). At the committee meeting held in January 1991, it was suggested that “it was time to review direction of GIG and to address major issues” (GIG 1991a). GIG first hired a fundraising officer, but his contract was terminated soon as he was not very successful. Then in 1992, members of the management committee learnt that, unbeknown to them, Hunt had suggested GIG might offer a full-time contract to Dalby, who had just left the Association to Combat Huntington’s Chorea (GIG 1992a). This incident made some committee members think that the organization needed “an independent director.” Dalby was tasked to conduct a survey to determine the areas on which the member organizations wanted GIG to prioritize in its work (GIG 1992b), and the result suggested that it should focus on policy-related activities (GIG 1992c). On that basis, the job description for directorship was produced, and the job was advertised in a nationwide newspaper *the Guardian* in January 1993. A few months later, Kent, who previously worked for a city council and a large charity organization on disability issues, joined the organization. While he only had basic knowledge of genetics, the management committee endorsed his vision to prioritize policy issues concerning the entire community of patients with genetic disorders (interviews UK01 and UK02).

Another important development was the creation of the European Alliance of Genetic Support Groups in 1992. Ysbrand Poortman, who founded the Dutch umbrella organization for genetic disorders called VSOP, led its creation in response to political integration in Europe. In 1991, patient organizations were invited for the first time to the European Society of Human Genetics (ESHG) annual congress, and there a preliminary meeting to consider forming a pan-European organization was held (interview NL01; Poortman 1999). GIG initially declined the invitation to join (GIG 1991b), but later overturned its decision and became a founding member when it was officially established at the ESHG meeting in Copenhagen. Kent saw the importance of this European network, and served on its management committee. His close working with European colleagues later paid off, offering him opportunities to represent patients in major European initiatives, including the European Platform for Patients’ Organisations, Science and Industry and the European Medicine Agency’s Committee for Orphan Medicinal Products, which both enhanced the credibility and importance of GIG in the UK.

The occasions that GIG represented patients’ voice also increased within the country since the mid-1990s. When the CGS Working Party published its report concerning predictive genetic testing on children after identification of the gene responsible for Huntington disease (Clarke 1994), GIG quickly published its response (Dalby 1995). And when the Advisory Committee on Genetic Testing was formed, in response to a 1995 House of Commons Science and Technology Select Committee report on Human Genetics that emphasized the need to involve lay members (Harper 1995; House of Commons Science and Technology Committee 1995), Lavery became a member as both director of the MPS Society and GIG’s founding member. After the Committee was absorbed into the Human Genetics Commission in 1999, Kent served on it. In 2002, GIG also became a partner of five out of six Genetic Knowledge Parks, which were multi-sector-collaboration initiatives to foster breakthroughs in genetics led by the government (GIG 2002). Because of the increased importance of medical genetics in medicine and increased demand for patient involvement in policy discussion in the early twenty-first century, the challenge for GIG shifted from finding opportunities to make its voice heard to ensuring its time and resources were spent on the right opportunities (interview UK01).

## Discussion

In the preceding sections, I have described the formation and evolution, from the 1980s to the early 2000s, of two genetic umbrella organizations: the AGSG in the USA and GIG in the UK. Specifically, I have sought to capture the very different origins and trajectories of the two organizations. We can understand these trajectories, I would argue, as embodying two rather different citizenship projects and forms of genetic citizenship – including different understandings of the rights and responsibilities that membership of those organizations entailed.

In both the USA and the UK, those affected by genetic disorders experienced and exercised genetic citizenship primarily through membership and participation in disease-specific organizations. In the USA, however, the AGSG’s early activities were primarily aimed at reorienting the activities of such organizations towards shaping and delivering genetic services in ways that had not previously been prioritized by clinical geneticists and other professionals. It did so by mobilizing their members’ experiential knowledge of living with particular genetic disorders, by creating opportunities for sharing organizational and other skills across those organizations, and by fostering an appreciation of what the members of different organization had in common. In so doing, it did much to build a common citizenship of genetic activism and service that transcended individual organizations.

Membership of that community remained largely confined to patients and families affected by genetic disorders, however. Medical professionals commonly endorsed, supported and helped to promote the AGSG and its activities – but they did so, on the whole, as friendly neighbors rather than community members. The roles of patient organizations and medical professionals were thus complementary rather than collaborative, drawing on their different knowledges and skills to deliver different if mutually supportive kinds of care. That complementarity was a constant theme in Joan O. Weiss’s vision of the role of the AGSG and its member organizations (e.g. Black and Weiss 1988; Macketa and Weiss 1994; Weiss 1989). Meanwhile, the interaction between patients and professionals commonly involved an element of negotiation over the kinds of services they would each offer. Indeed, negotiation with medical and scientific professionals would become even more important in the renamed Genetic Alliance’s work in the early 2000s, as the organization reoriented its activities to focus on leveraging control of its members’ biological resources as means of determining the direction and purpose of genetic research.

In contrast, GIG was emerging in the UK as a very different kind of umbrella organization, representing a very different kind of genetic citizenship. Rather than seeking to inform or coordinate the activities of its member organizations, it was created to provide them with representation in national policy processes. As such, it served in effect to claim and assert an additional dimension of citizenship to those already exercised by individual disease organizations. To that end, it provided a forum for its member organizations to formulate and express a shared view on matters of policy concern. Furthermore, it did so in close contact with medical geneticists and other professionals. Underpinning Harris’ original call for disease organizations to formulate a common position on matters of policy was an assumption that that position would be in broad alignment with the views of medical geneticists. GIG’s constitution, with its medical advisory board made up of some of the country’s most prominent geneticists, helped to realize that assumption. In giving a representative dimension to genetic citizenship, GIG not only drew on its members’ expertise by experience, but also on the views of medical experts. As my interviewees put it, where the American organization negotiated with the professionals, the British cooperated with them (interviews US08 and UK01).

The different constitutions of the two umbrella organizations, and the different forms of genetic citizenship they embodied, were partly due to the visions and personalities of their founders. Yet they were also shaped by the different ways in which genetic services developed in the USA and the UK, and the different needs and opportunities they presented to patients and families affected by genetic disorders. In both countries, genetic services initially grew from the clinical and research interests of a handful of pioneer medical geneticists. From there, however, they followed rather different trajectories.

In the USA, genetic services were shaped by “the model of business enterprises” (Mulvihill, Walters, and Wertz 1989, 421), leading to a diversity of clinics that varied widely in the kinds of services they offered and the fees they charged. Under Weiss’s leadership, the AGSG functioned primarily as a platform through which patient support groups learnt how to mobilize their members’ knowledge and experience with the dual aim of bringing existing services more closely in line with their own needs, and establishing their own alternative support services where professional provision fell short. In a context of predominantly commercial healthcare, the AGSG was animated chiefly by a desire to advance its members’ rights as consumers of medical services. Sharon Terry’s leadership from the 2000s, and especially her championing of the organization’s own biobank, effectively extended this approach by using members’ biological inheritance as a resource to trade in return for an influence on the direction of research and the delivery of new treatments (Heath, Rapp, and Taussig 2004; Taussig 2005, 2007; see also Terry and Davidson 2000).

In contrast, genetic services in the UK developed under the National Health Service, and were shaped by the universalistic expectation that “any innovation of proven value should be available to all patients” (Harris and Wertz 1989, 409). In practice, however, genetic services were initially a low priority in the major reorganization of the NHS that took place during the 1970s, and medical geneticists’ ideas about what services offered “proven value” did not necessarily coincide with the views of those affected by genetic disorders, as was apparent in debates over testing for Huntington disease. Realization of this resulted in Harris’s call for a united voice to represent genetic patients in policy debates, and the decision by representatives of a number of genetic diseases organizations to establish GIG for just that purpose. Founded, in the words of its first chairperson Ann Hunt, as “a forum to publicize” the needs of the genetic disease community (GIG 2006, 17), the organization has since worked with medical geneticists to ensure the availability of high-quality genetics services across the country (e.g. Raeburn, Kent, and Gillott 1997; see also GIG 2006; Kerr 2003).

Genetic umbrella organizations in the USA and the UK thus pursued rather different citizenship projects, reflecting the divergent structural and cultural organizations of medical genetics in the two countries – what Parthasarathy (2007) calls the distinct “toolkits” of genetic medicine – which in turn presented the AGSG and GIG with different demands and opportunities for action. In this respect, the forms of active, participatory citizenship that most attracted the attention of social scientists in the mid-2000s more closely resembles that championed by the AGSG, characterized by its emphasis on the collective efforts of patients and their families to influence the work of medical professionals and healthcare providers (e.g. Heath, Rapp, and Taussig 2004; Novas 2006, 2007, 2008; Rose and Novas 2005; Taussig 2005, 2007). This was a form of genetic citizenship that focused on the rights of patients both as consumers of professional services and as owners of their own biological materials, within an entrepreneurial and largely commercial healthcare system. In contrast, the form of genetic citizenship championed by GIG in the UK focused on patients’ right to be represented in policy decision about the development of a universal health service.

Of course, these differences were never clear cut or absolute, and there was considerable overlap. In both countries, genetic citizenship drew heavily on patients’ own experiences and the expertise that entailed. And in both countries, the multiplicity of different disease organizations sustained a diversity of initiatives aimed at securing the best services for patients, with which umbrella organizations inevitably became entangled. Not least, in both settings, the activity of umbrella organizations and their constituent disease organizations raise questions concerning any organization’s capacity and legitimacy to represent the interests of its members (Jongsma *et al*. 2018; Baggott and Jones 2018). But as this article has sought to demonstrate, the differences are as illuminating as the commonalities, and deserve to be taken seriously in academic efforts to analyze the forms and meanings of genetic citizenship.
